# Impact of electrode geometry and thickness on planar on-chip microsupercapacitors

**DOI:** 10.1039/d0ra05488g

**Published:** 2020-08-26

**Authors:** Agin Vyas, Kejian Wang, Qi Li, Amin M. Saleem, Maria Bylund, Rickard Andersson, Vincent Desmaris, Anderson Smith, Per Lundgren, Peter Enoksson

**Affiliations:** Micro- and Nano Systems Group, EMSL, MC2, Chalmers University of Technology Kemivagen 9 41296 Gothenburg Sweden agin@chalmers.se; Smoltek AB Gothenburg Sweden

## Abstract

We report an assessment of the influence of both finger geometry and vertically-oriented carbon nanofiber lengths in planar micro-supercapacitors. Increasing the finger number leads to an up-scaling in areal power densities, which increases with scan rate. Growing the nanofibers longer, however, does not lead to a proportional growth in capacitance, proposedly related to limited ion penetration of the electrode.

## Abstract 2

We present an in-depth analysis of the impact of geometry and carbon nanofiber thickness on CMOS compatible microsupercapacitor performance.

## Graphical abstract

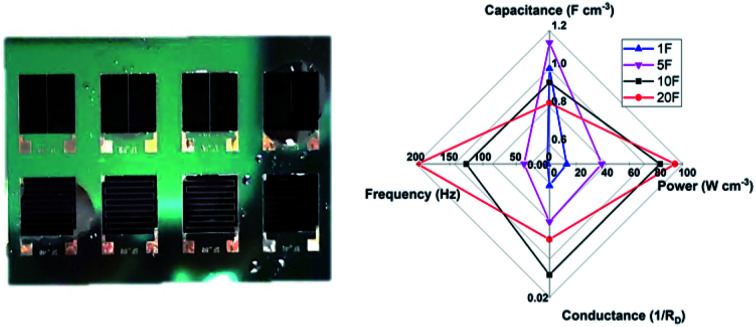

The technological trends have been moving beyond Moore's law in recent years, with an increasing focus on device performance in a miniature package. Applications such as the Internet of Things (IoT)^1^ and integrated circuit (IC) processors^2^ can benefit from a miniaturized power supply which can be incorporated with their on-chip components. In order to realize a high-performance on-chip power supply, integration of electrochemical capacitors, also known as supercapacitors, with energy harvesting sources, has become a robust solution candidate.^3^

Micro-supercapacitors (MSCs) are miniaturized energy storage units that can use the principle of electric double layers at their electrode–electrolyte interface for charge storage. Due to the electrode's high surface area, the micro-supercapacitors demonstrate a high capacitance while possessing high power density and long cycle lifetime. These characteristics make them a viable solution for on-chip power supplies for integrated sensors, RF filters, or decoupling capacitors for integrated circuit (IC) assembly provided that they can be fabricated through a complementary-metal-oxide-semiconductor (CMOS) compatible technology.^4,5^ Chemical vapor deposition of carbon electrodes can produce several MSCs on a single wafer with high yield and uniformity between devices.^6–8^ This technique is used to grow several high surface area, highly porous carbon materials such as carbon nanotubes (CNTs),^9^ reduced graphene-oxide (rGO),^10^ vertically aligned carbon nanosheets (VACNS),^11^ vertical graphene (VG), carbon onions,^12^ and carbon nanofibers (CNFs).^11^ Among these, carbon nanofibers can be grown at highly CMOS compatible temperatures of 390 °C and 550 °C, which can be viable in some cases where aluminium is not used.^13^ The CNF is especially suited for integration onto a CMOS chip due to selective growth at the desired location at temperatures within the CMOS tolerance window.^14^ Due to the reliability of the growth mechanism, high yield, and consistant performance, CVD grown CNFs can be grown for different applications while meeting their specific charge storage and power delivery demands. Moreover, the properties of the MSCs can be modulated based on their geometric designs. MSCs can be fabricated as stacked electrodes or with planar current collectors. The former structure is severely limited in performance due to long ion diffusion and charge transportation paths. The planar architecture decreases the ion diffusion resistance by shortening transport lengths. Previous studies have been conducted pertaining to the influence of finger geometry on the performance of on-chip electrochemical capacitors using pseudocapacitive electrode materials such as RuO_2_,^15^ polyaniline MnO_2_,^16^ layer-by-layer deposited rGO,^17^ CNTs with PANI,^18^ and multiwalled CNTs in polymeric cavities.^19^ Although these results establish an increase in power density of the device with increasing number of fingers, the effects on capacitance, device resistance and cut-off frequency still require considerable attention. Similarly, the effects of thickness have been studied^18,20,21^ when the electrodes were used as sheets for coil cells. There have been studies relating on performance of CNF electrodes grown from CVD by Saleem^22^ and Andersson^9^ on Ni and Cu electrodes. Both the studies suggest that reducing the spacing between interdigitated fingers reduces the discharging time. However, on comparing their device performances based on thickness, they demonstrate nearly equivalent areal capacitances even though one of them has 5 μm long CNF, while the other has 17 μm. Therefore, there is need to study the effects of CNF lengths on electrode performance.

In this manuscript, we discuss the effects of the interdigitated fingers on the device capacitance, power density, resistance, and device cut-off frequency for CVD grown CNFs through the experimental analysis of multiple MSCs with one (1F), five (5F), ten (10F), and twenty (20F) fingers of a fixed length (4.7 mm) and individual finger widths as 4.7 mm, 0.42 mm, 0.2 mm, and 0.08 mm respectively. The outer length (*l*) and width (*w*) of the devices are kept constant at 4.78 mm and 4 mm respectively, with the exception of a width of 2.2 mm for the 1F device. The 1F device has 2 current collector pads separated by a distance *w*_i_ between them. The entire electrode footprint area, *A*, is calculated as *A* = *w* × (*w*_*n*_ + *w*_i_) × 2*n*, where *w*_*n*_ is a single finger's width and the number of fingers on a device is defined by *n*. The volume (*V*) is *A* × *t* where *t* is the thickness. The device performances such as areal capacitance (*C*_A_), energy and power densities are calculated in accordance with the performance metrics described by Kyeremateng *et al.*^23^ The metrics described in the article are translated to relatable application-based units. The 1F and 5F have footprint areas of 0.21 cm^2^ and 0.20 cm^2^ respectively while the 10F and 20F have 0.15 cm^2^ and 0.14 cm^2^ respectively. We highlight the issues relating to trade-off of increasing the CNF electrode thickness and capacitance with device frequency and resistance by growing CNFs of lengths 3 μm, 5 μm, 8 μm, and 14 μm. In order to make the device closer to CMOS compatibility we have used palladium as current collectors, which has recently been used in MSC fabrication and IC compatible processes.^24^

The schematic in Fig. 1(a) shows the fabrication process. A 2′′ silicon (100) wafer, of 280 μm thickness was used as a substrate. A 400 nm thermal SiO_2_ was grown on the 2′′ substrate (Fig. 1(a-i)). The Pd/Ti (100/20 nm) current collectors and catalyst were lifted off on the substrate using a positive photoresist. The Si/SiO_2_ substrate was then diced into individual chips. Dicing is followed by a backside metallization (Ti/TiN) (30/100 nm) for improved thermal contact during the CVD process (Fig. 1(a-ii)). The growth mechanism of CNF relies on the de-wetting of metal catalyst droplets under proper adequate plasma conditions. Details on the mechanisms and resulting structures can be found in Desmaris *et al.*^25^ and the analysis of typical CNFs grown under the same conditions have been reported in Saleem *et al.*^26^ The CNFs are grown onto these nanoparticles at temperatures of 390 °C and 550 °C at varying times for a growth of 3 μm, 5 μm, 8 μm, 12 μm and 14 μm (estimated using Veeco Dektak Profiler) using a mixture of acetylene and ammonia in direct current plasma enhanced CVD (Fig. 1(a-iii)). 1-Ethyl-3-methylimidazolium bis-(trifluoromethylsulfonyl)imide (EMIM-TFSI) was chosen as the electrolyte due to a higher operational voltage window than aqueous electrolytes and due to its superior conductivity and electrochemical and thermal stability over other ionic liquid electrolytes (Fig. 1(a-iv)). The fabricated devices with the electrolyte at room temperature are shown in Fig. 1(f). SEM images confirm that the CNFs are grown vertically aligned with the individual CNF strands in clusters, with uniform lengths over each individual device as shown in Fig. 1(b–e). Fig. 1(g) shows a schematic representation of the finger geometry of the MSC current collectors.

**Fig. 1:**
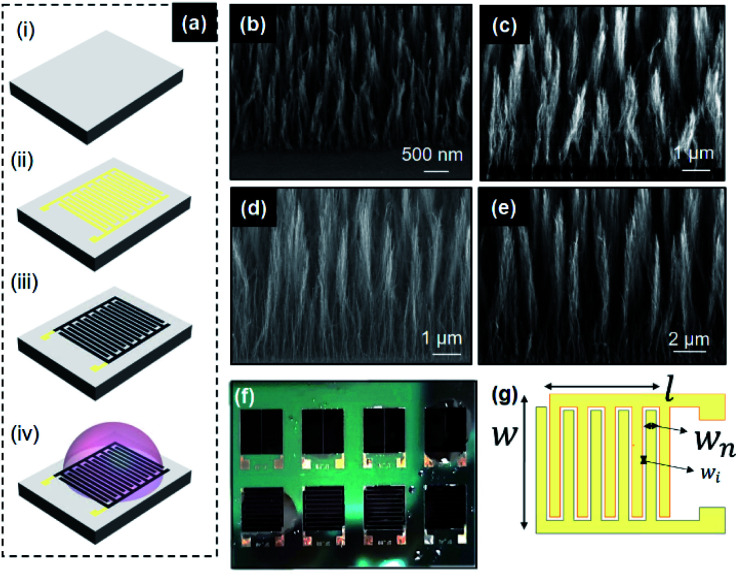
(a) Schematic fabrication process of CVD grown CNF on Si/SiO_2_ dies. Scanning electron micrographs of various grown-CNF thickness (b) 3 μm, (c) 5 μm, (d) 12 μm, and (e) 14 μm at different magnifications. (f) Optical micrographs of the fabricated MSCs. (g) Top view of the schematic MSC device.

Once fabricated, the devices were measured at room temperature in open atmospheric conditions in EMIM-TFSI solution at a voltage range of 0–1 V. The cyclic voltammetry measurements were carried out at constant scan rates of 100–5000 mV s^−1^. The capacitance of the devices for charging and discharging was calculated and later averaged from Fig. 2(a) as *C* = ∫*I*d*t*/Δ*V* where *C* is the total capacitance, *I* is the current in the device, *t* time, and Δ*V* as the voltage window for the electrolyte, taken at 1 V in this work. The galvanostatic charge–discharge measurements were operated under a constant current from 1–100 μA cm^−2^. The capacitance through these experiments is calculated as *C* = *I* × *t*_d_/(Δ*V* − *V*_drop_), where *I* is the current density, *t*_d_ is the discharging time and *V*_drop_ is the voltage drop at the charge discharge switching time. The energy density is calculated as *E*_*S*_ = *CV*^2^/(2*S*) where *S* is equal to *A* or *V* for areal and volumetric density respectively. The power density is calculated as *V*^2^/(4*R*_D_*S*). Impedance spectroscopy (EIS) was measured in a frequency range of 10 mHz to 10 MHz at a *V*_RMS_ of 5 mV. The characteristic frequency (*f*_k_) also known as the knee-point frequency is calculated at −45° phase angle. It is used to calculate the time required to discharge with more than 50% efficiency.^27^

**Fig. 2:**
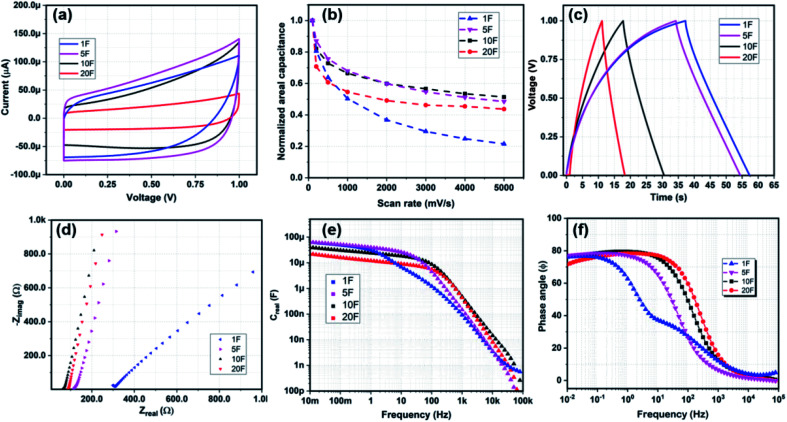
(a) Cyclic voltammetry at 1000 mV s^−1^, (b) normalized areal capacitance *vs.* scan rate. (c) Chronopotentiometric charge–discharge cycles at constant current density of 5 μA cm^−2^. (d) Nyquist plot for potentiostatic impedance spectroscopy. (e) Bode plot of real device capacitance (*C*_real_) and (f) *Z*_phz_*vs.* frequency.

Fig. 2 shows the electrochemical results for MSCs with a thickness of 3 μm for all the devices. The thickness is selected based on the uniformity of the grown CNF heights as these devices were fabricated on the same silicon die. These results also fairly represent the trends in devices with increasing electrode thickness as the impact of thickness is not substantial on the device characteristics, as we will show. The scan rates and frequencies were selected to simulate expected output signals from a power management unit and an energy harvester for an on-chip power supply for wireless sensor networks. At low scan-rates below 100 mV s^−1^ or low small signal frequencies, the capacitance decreases with increasing finger number as *A* decreases with more fingers. However, at scan-rates higher than 100 mV s^−1^, the areal capacitance of 5F is highest, followed by 1F, 10F, and 20F. 20F device demonstrates the lowest capacitance as it has the least electrode material on its current collectors. Comparing the normalized areal capacitance (Fig. 2(b)) over scan rate for different finger structure, we find that the 5F, 10F, and 20F devices show similar saturation trends at high scan rate, whereas the 1F device demonstrates a poor rate capability of merely 20%. In Fig. 2(c), we see the galvanostatic charge–discharge curves for all the devices. The 1F takes a longer time to charge completely at 1 μA cm^−2^ to 1 V as it has the largest electrode area. It is interesting to note here that the charging curves for 1F and 5F are rather curved compared to 10F and 20F. The device resistance (*R*_D_) and Warburg impedance is observed in Fig. 2(e) that shows the EIS for the devices. The intercept at the *Z*_real_ axis displays the *R*_D_, which decreases incrementally with increasing number of fingers. This is in correlation to the charge–discharge measurements in Fig. 2(c) where the 1F and 5F curves take a long time to charge. The slope of the Nyquist plot for the *Z*_real_ and −*Z*_imag_ determines the Warburg impedance of the device, with a vertical slope suggesting an ideal porous behaviour. Fig. 2(d) shows near equivalent behaviour for the finger devices 5F, 10F, and 20F. However, for the 1F, the trends suggest that there is an additional phase element which can be due to longer ion-transportation distance at lower frequencies that is contributing to the total impedance. Fig. 2(e and f) shows the maximum real capacitance (*C*_real_) and phase behaviour (*ϕ*) of the devices over a wide frequency spectrum. The finger devices have an equivalent characteristic behaviour in terms of capacitance (*C*_real_), *f*_k_, and phase constant (*ϕ*), changing slightly with the number of fingers; whereas the 1F device displays a behaviour with high *R*_D_, low *f*_k_ and lower capacitance at frequencies higher than 1 Hz. It also appears that at low frequency the capacitance for the 1F and 5F devices is about three times higher than for the 20F, in part owing to their larger effective area. However, as we go towards high frequencies, the 10F and 20F devices overshadow the capacitive advantage of 1F and 5F devices owing to a lower resistive component. Interestingly, the 1F device would have had more similar features compared to the finger devices were it not for the apparent additional phase element acting at low frequency between 10–100 Hz, visible in Fig. 2(f).

Fig. 3 is an assembly of results from all the different devices measured and clustered for a generic device understanding. Fig. 3(a) shows the variation of *R*_D_ and *f*_k_ over the number of fingers. The device resistance for 1F is substantially higher than that of the finger devices. The characteristic frequency, *f*_k_, is generally dependent on the geometry of the device and on the electrode–electrolyte interaction. Therefore, observing a decrease in *f*_k_ with increasing thickness is a sign of marginal increase in *R*_D_ and possibly reduced ion-penetration at large thicknesses. Fig. 3(b) shows the energy and power densities of the devices for 3 μm electrode thickness. As expected, the 1F and the 5F devices demonstrate a higher energy density than the 10F and 20F while the latter demonstrate a higher power density. In Fig. 3(c), with increasing CNF thickness, the volumetric capacitance of the devices decreases over all fingers. It seems like there could be a slight peak at 5F, indicating that for an increase in the number of fingers some active volume might be lost. Fig. 3(d and e) shows the variation of total capacitance, areal energy density and total and areal power densities of the devices over increasing CNF thickness at 5 μA cm^−2^ and 10 mHz at 0.1 V respectively. The main conclusion is that the CNF thickness shows no consistent effects on the capacitance/energy or power in general. From Fig. 3(a), however we can speculate that for a high number of fingers there is an effect on frequency with thickness. Since, power should scale with energy, the minimum power at 12 μm and the maximum energy at 12 μm might indicate that there is some kind effect of slowing down the response at 12 μm. At 12 μm and 14 μm, the device power is approximately half of the maximum achievable power at 5 μm thicknesses. Since it would be strange with 12 μm being significantly different than 14 μm, we can say that the results in Fig. 3(d and e) mirror the reduced frequency with thickness shown in Fig. 3(a). Fig. 3(f) represents an bar plot of the changing areal capacitance of the 1F and 5F devices over a range of current density inputs. Finally, in Fig. 4(a), we observe the normalized radar plot for the main parameters for device performance evaluation. It shows that considering all these characteristics, the 20F device performs the best in terms of the area inside the triangular representation, irrespective of the thickness of the electrodes.

**Fig. 3:**
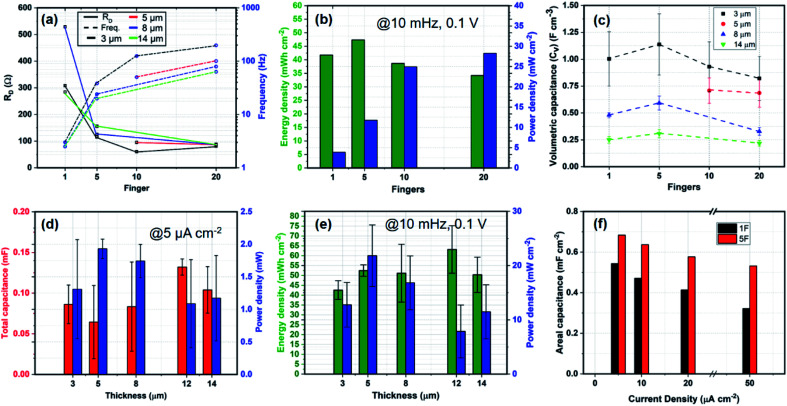
(a) Device resistance (*R*_D_) and knee point frequency (*f*_k_) *vs.* fingers of all the fabricated devices. The dashed lines show the trends captured for *R*_D_ and *f*_k_ for varying CNF thickness. (b and d) Areal energy and power densities of the MSC devices. (c) Volumetric capacitance of the MSC devices of different thicknesses with increasing number of fingers. (d) Total device capacitance and power with respect to thickness of the CNF. (e) Energy/power density and areal capacitance *vs.* thickness of CNF. (f) Areal capacitance *vs.* current density of 1F and 5F MSCs.

**Fig. 4:**
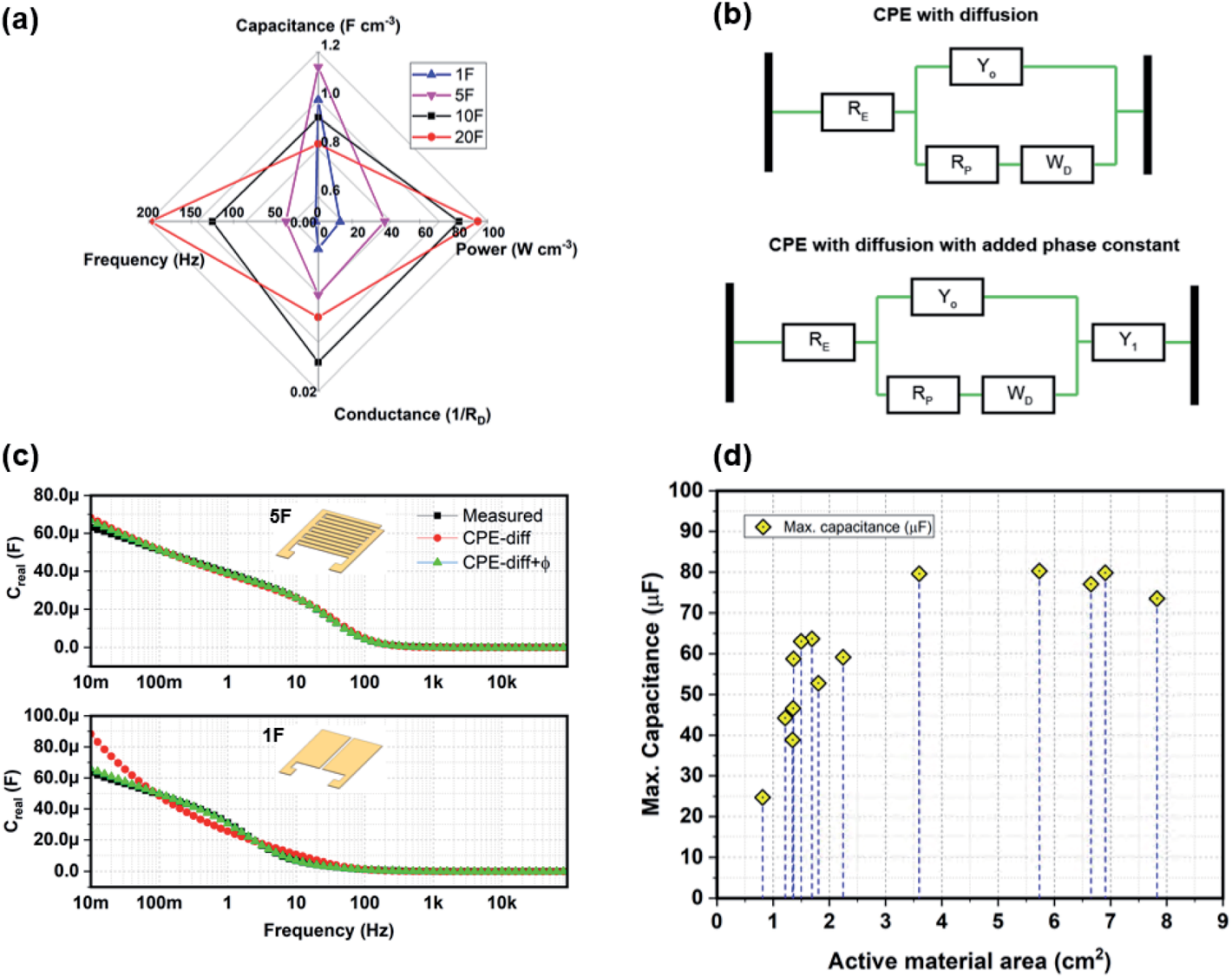
(a) Representative radar plot of volumetric capacitance, power density, conductance, and frequency of 1F, 5F, 10F, and 20F MSCs, in this case, for 3 μm CNF thickness. (b) Equivalent circuit models used to analyse MSC electrochemical performance. (c) Bode plot for the *C*_real_*vs.* frequency plot for the 5F an 1F MSC. The devices are modelled with a CPE with diffusion model, (d) maximum capacitance exhibited by the MSCs over a range of active material area.

We have now discussed the fabrication, characterization, and results of CVD grown CNF based MSCs with different geometric configurations and electrode thicknesses. The first major highlight of increasing the number of interdigitated fingers while keeping the device area constant is an improvement in the power density of the device. The systematic increase in power density in the higher finger devices compared to 1F can be elucidated through two possible explanations – firstly, reduction in ion-diffusion path length leading to reduced device resistance and secondly, increasing the circumferential area of electrode–electrolyte interaction. Pech *et al.*^15^ have proposed the electrolyte resistance as one of the most influential factors in determining *R*_D_, the total resistance of the device. As the number of fingers increases, the electrolyte resistance decreases due to a reduced ion-transport distance, resulting in a reduced cell constant for the device. The cell constant is a parameter that describes the proportionality between the *R*_D_ and *R*_E_, where the latter is the electrolyte resistance. Assuming a fixed electrode area, the maximum ion diffusion path in an MSC device would be the width of the electrode (*w*_*n*_) plus the distance between the fingers (*w*_i_). Since 1F has the largest width among all the devices, it leads to higher ion diffusion length. 5F, 10F, and 20F devices have widths approximately ∼0.05 × *w*_*n*_ of 1F. An increased width results in an increase in *R*_D_, which is a combination of *R*_E_, the electrolyte resistance and *R*_ct_, the charge transfer resistance. As there are no charge transfer reactions expected in the electric double layer system, *R*_E_ is the device component that determines *R*_D_. To elucidate this further, the EIS behaviour of the MSCs was observed through equivalent circuit modelling. The circuit models are shown in Fig. 4(b). The first model comprises a constant phase element (CPE-diff) with diffusion, previously explained in Bisquert.^28^ The second model is CPE with diffusion with an added constant phase element in series, an extra delay due to diffusive transport in the electrode. It is used to describe the behaviour of the 1F devices. In both the figures, *R*_E,P_ are the equivalent series and parasitic resistances, *Y*_0,1_ are the constant phase elements, and *W*_D_ is Warburg impedance. Fig. 4(c) shows the fitted model by using the simplex method^29^ to optimize the parameters with the measured EIS results for the 1F and 5F device (considered an equivalent to the remaining finger devices, see Fig. 2(e and f)). The finger devices 5F, 10F, and 20F fit CPE-diff with an *R*^2^ value of 0.997. The 1F device demonstrated *R*^2^ = 0.953 with the same model. The fit suggests that 1F requires an additional constant phase element to achieve the same aspect of fit as observable in the frequency range of 1–10 Hz. The 1F device gives a measure of *R*^2^ = 0.999 with CPE-diff with added phase element, with a similar goodness of fit for 5F. The fitted values for *R*_E_ are consistent with what can be extrapolated directly in from the *Z*_real_ axis intercept in the EIS measurement. The Warburg impedance and the parasitic resistance of the 1F device are much higher than 5F, 10F, and 20F devices. In case of *W*_D_, the 1F device possesses a value of Warburg coefficient as 6.3 × 10^−12^ S s^1/2^, while the 5F shows 4.8 × 10^−10^ S s^1/2^. A higher coefficient value signifies increased diffusivity.^30^ In high finger numbers, the Warburg coefficient is small since diffusing electrolyte ions do not have to travel far to form a double-layer, while with single finger electrodes, the ions have to diffuse further, increasing the Warburg impedance.^31^ Coefficient of porosity closer to unity is another measure of an ideal capacitive behaviour. The 1F device demonstrates a porosity of 0.74 while 5F shows 0.86. Similarly, for parasitic resistance, the diffusion length in 1F device is much larger than the finger devices. Therefore, according to the model, 1F devices demonstrate a parasitic resistance of 128 Ω, while the 5F, 10F, and 20F devices show a parasitic resistance of 23 Ω, 11 Ω and 8.7 Ω respectively. These results demonstrate that increasing the number of fingers reduces the ion transportation distance, which in turn, leads to improved conductance.

The second major inference from the results is that increasing the CNF thickness is not necessarily the optimal way to improve the device capacitance. It is suggested that increasing the CNF thickness beyond 12 μm, not only reduces the power density of the device, but it does not affect the overall device capacitance. Fig. 4(c) is another representation of the maximum capacitance exhibited by the MSC devices over the total active electrode area. The active electrode area for CNF-based MSC was previously evaluated by Saleem *et al.*^25^ The total surface area of a collection of CNFs can be calculated by *N*(2π*rl* + π*r*^2^); where *N* is the approximate number of CNF fibers in a given area, *r* is the radius of a single CNF fiber approximated as 100 nm, and *l* is the length of the CNF grown on the substrate. In the figure, the maximum capacitance of the CNF displays a constant capacity despite increasing CNF lengths beyond 12 μm. Previously, Anderson *et al.*^9^ fabricated CNF based MSC devices with 17 μm thick electrodes. Their MSCs had a finger width of 100 μm with a gap of 30–50 μm between them. The results for those devices directly compare to the 20F device which has a width of 80 μm and spacing of 40–60 μm. On inspection, their devices demonstrated an areal capacitance of 0.41 mF cm^−2^, which is similar to the value achieved with 20F at 14 μm thickness. Similar inferences from increasing the electrode thickness have been observed in different materials such as CNT/PANI electrodes^18^ and pseudocapacitive electrodes such as hydrous and electrodeposited RuO_2_ electrodes.^15^ The authors have suggested that the reduction in volumetric capacitance can be attributed to a decrease in the ion-penetration in the compact inner layer. One example of such an effect can be seen in Fig. 3(c) that shows the volumetric capacitance of the devices over varying fingers and thicknesses. The volumetric capacitance of the device, which should be constant as it is ideally a material dependent property, decreases with increasing thickness across all fingers. Another explanation for the reduced volumetric capacitance can be the reduced ion penetration at high thicknesses. Reduced areal capacitance while increasing CNF thickness can be due to the poor electrolyte–CNF interaction as suggested by Saleem *et al.*^15^ In our case, we observe an increase in device resistance with increasing thickness through EIS measurements, suggesting that the CNF parts farther from the applied potential plane are unable to bind electrolyte ions. This should correlate with the most part of the extended CNFs being “screened” from taking part in the ion accumulation, *i.e.* we would not be limited by ion penetration, but by the CNF active area only being the CNF part closest to the surface. These are two opposing theories that can be explored further in future work. The aspect of improved electrolyte penetration can be studied in the future through cyclic stability studies over different thicknesses. This would provide an interesting insight into the mechanisms of interaction between CNF and EMIM-TFSI as an electrolyte also. The issue of stability of the electrolyte can also be addressed in future studies, including considering hermetic packaging solutions.

The key results for cell design show that 5F devices have the highest energy densities in a 1 cm^2^ device area with the maximum achievable energy of 0.54 mF cm^−2^ at 12 μm thickness. The 20F device with 5 μm thickness demonstrated the largest power density of 27 mW cm^−2^ with a capacitance of 0.38 mF cm^−2^. If we keep in mind the trade-off with energy and power density due to the geometric effect on the amount of electrode material, the 20F device performs the best in terms of the area inside the triangular representation, irrespective of the thickness of the electrodes, shown in Fig. 3(f). It is a combination of high device capacitance, followed by a large power density to operationalize miniaturized wireless sensors and transceivers while having a high *f*_k_ and low *R*_D_. Therefore, the best performing device in our set, combining high capacitance, power density and improved diffusion coefficient through low *R*_D_ would be a 10F device with 12 μm thick CNF electrodes while having a spacing of 40 μm between the interdigitated fingers.

## Conclusions

We have reported the investigation on the fabrication of PECVD grown CNF based MSCs on CMOS compatible current collectors with different thicknesses and geometric designs. Among all the devices, the devices with more than one finger on each current collector demonstrated a decrease in areal capacitance due to reduced active electrode footprint during the low frequency response. The power density of the devices, irrespective of the electrode thickness, increases with increasing number of fingers owing to lower device resistance. Overall, while including the effects of device resistance, the MSC with 10 fingers displays the best overall performance while incurring the lowest trade-off costs. The thickness of the electrode has a very small impact on the capacitance, characteristic frequency, and resistance. In the future, studies need to be conducted to further investigate the weak impact of electrode thickness, the effect of different electrolytes combined with CNF, and the influence of the electrode electrolyte wettability.

## Conflicts of interest

There are no conflicts to declare.

## Supplementary Material
